# Is Serum Vitamin D Associated with Depression or Anxiety in Ante- and Postnatal Adult Women? A Systematic Review with Meta-Analysis

**DOI:** 10.3390/nu16213648

**Published:** 2024-10-26

**Authors:** Luis Otávio Lobo Centeno, Matheus dos Santos Fernandez, Francisco Wilker Mustafa Gomes Muniz, Aline Longoni, Adriano Martimbianco de Assis

**Affiliations:** 1 Graduate Program in Health and Behavior, Catholic University of Pelotas (UCPel), Pelotas 96015-560, RS, Brazil; luis.centeno@sou.ucpel.edu.br (L.O.L.C.); aline.longoni@ucpel.edu.br (A.L.); 2 Graduate Program in Dentistry, Federal University of Pelotas (UFPel), Pelotas 96015-560, RS, Brazil; matheus.fernandez@ufpel.edu.br (M.d.S.F.); muniz.fwmg@ufpel.edu.br (F.W.M.G.M.)

**Keywords:** depression, anxiety, pregnancy, vitamin D, postpartum, puerperal cycle

## Abstract

Background/Objectives: To collect evidence from studies that explored the associations between serum vitamin D (25[OH]D) concentrations/status and the presence of depressive/anxiety symptoms in the ante- and/or postnatal periods (PROSPERO-CRD42023390895). Methods: Studies that assessed serum 25[OH]D concentrations in adult women during the ante/postnatal periods and those that used valid instruments to identify the experience/severity of depressive/anxiety symptoms were included. Independent researchers performed the identification/selection of studies, data extraction, risk of bias (RoB) assessment, and bibliometric analysis steps. Results: Of the total of 6769 eligible records, 15 cohort studies [high (*n* = 3), moderate (*n* = 7), and low (*n* = 5) RoB], nine cross-sectional studies [moderate (*n* = 3) and low (*n* = 6) RoB], and one case-control study [moderate RoB] were included (*n* = 25). Depression (*n* = 24) and anxiety (*n* = 4) symptoms were assessed. A significant difference in antenatal serum 25[OH]D concentrations between the groups of women with and without depression was identified (mean difference: −4.63 ng/mL; 95% confidence interval [95% CI]: −8.88; −0.38). Postnatal serum 25[OH]D concentrations were found to be, on average, −2.36 ng/mL (95% CI: −4.59; −0.14) lower in women with postnatal depression than in those without. Maternal antenatal anxiety was associated with significantly lower concentrations/deficiency of 25[OH]D in only one included study. Conclusions: Based on very low/low-quality evidence, it was observed that reduced serum 25[OH]D concentrations in the ante- and postnatal period are associated with the presence of ante- and postnatal depressive symptoms, respectively. Low/deficient antenatal serum 25[OH]D concentrations may not be related to the presence of anxiety symptoms before childbirth. Well-designed longitudinal studies are needed to explore the estimated pooled effect of these associations.

## 1. Introduction

Vitamin D (25[OH]D) is a fat-soluble vitamin found in several animal- and plant-based products and fortified foods and is also made endogenously in the human body [1]. Serum 25[OH]D is used to assess the status of 25[OH]D. The Institute of Medicine defines 25[OH]D deficiency as a serum 25[OH]D concentration <20 ng/mL (50 nmol/L), 25[OH]D insufficiency as 20–30 ng/mL (50–75 nmol/L), and 25[OH]D adequacy as >30 ng/mL (75 nmol/L) [2]. The deficiency/insufficiency of 25[OH]D during the gestational period, in particular, is highly prevalent and represents an issue of relevance to public health [3,4]. Many factors can influence the 25[OH]D status of pregnant women, including latitude, season, diet, use of dietary supplements, time spent outdoors/sun, clothing habits, use of sunscreen and medications, body weight, ethnicity, and medical conditions [5,6,7,8]. Recently, a study with 34,417 pregnant women revealed that 9.9% of the population was severely 25[OH]D deficient, 60.1% was deficient, and 28.4% was insufficient; only 1.6% of the enrolled population reached an adequate level [9].

The 25[OH]D metabolism during pregnancy is different from that at any other time in human physiology. During this period, due to the need to supply calcium, the fetus requires serum maintenance of higher concentrations of the active form of 25[OH]D. Physiologically, 25[OH]D acts as a prohormone that plays an important role in calcium absorption, and its low concentration during pregnancy can predispose individuals to intrauterine growth restriction and adverse fetal/neonatal outcomes (e.g., abortion and low birth weight) [4,5,8]. Low serum 25[OH]D availability may also influence aspects of maternal health, increasing the risk of common mental disorders such as depression and anxiety [10,11].

Evidence shows that depression and anxiety are highly comorbid in the ante- and postnatal periods [12,13]. Several factors are predictors of these conditions in this population group at different times during pregnancy, such as sociodemographic and economic aspects (e.g., income, education, and ethnicity), previous history of mental illness, behavioral aspects (e.g., alcohol consumption and smoking), marital and social satisfaction, and negative cognitive styles [14,15,16]. Current efforts in the literature also indicate that different pathophysiological mechanisms are also related to the occurrence of depression and anxiety in pregnant women. In general, multiple hypotheses about the possible pathophysiological mechanisms of these mental disorders appear to be related to (I) HPA axis imbalance; (II) alterations in pro-inflammatory cytokines; and (III) changes in estrogen leading to a decrease in serotonin concentration [17]. These three pathways are related to cortisol secretion, impairing neural cells’ glutamatergic system and redox status [18,19,20,21].

In general, multiple hypotheses regarding the possible mechanisms of these mental disorders and 25[OH]D have been suggested. It is speculated that active 25[OH]D reverts the pathological action of glutamate increasing the efflux of Ca^2+^ in nerve cells and, therefore, contributes to alterations in neurotransmitters and behavioral changes [22,23,24]. Research has shown that 25[OH]D is an essential hormone in the synthesis of monoamines such as serotonin, noradrenaline, and dopamine [16,25]. Thus, 25[OH]D is involved in important brain processes (e.g., neuroimmunomodulation and brain development/neuroplasticity), which could indirectly impact mood regulation [26].

Previous systematic reviews published more than half a decade ago, and based on different methods of measuring and defining 25[OH]D concentrations, have explored the associations between 25[OH]D and depression in women during pregnancy who did not use an additional 25[OH]D supplementation [10,11,14,27,28]. Furthermore, another review concludes that, due to the small sample size, low quality, and high heterogeneity of the studies, the evidence currently available in the literature is insufficient to establish a role for 25(OH)D supplementation in the prevention or treatment of ante- and postnatal depression [29]. Estimates indicate that serum 25[OH]D concentrations <50 nmol/L are associated with a 3.67-fold (odds ratio [OR]: 3.67; 95% confidence interval 95% CI (95% CI): 1.72–7.85) increased risk of postnatal depression [10]. Other estimates have shown that high concentrations of 25[OH]D in the blood have a protective effect on maternal depression (OR: 0.49; 95% CI: 0.35–0.63) [27]. A few observational studies have also indicated that anxiety may not be associated with the serum level of 25[OH]D [30,31,32,33]. Although the previous evidence is useful for guiding public policies on maternal health care, it is crucial that the global assessment of serum 25[OH]D concentrations according to the diagnostic criteria for common mental disorders be carried out taking into account the period of data collection and the women’s pregnancy cycle. New epidemiological studies have been published in the last quadrennium (2020–2024) and a systematic update with recent findings can provide an up-to-date perspective on the relationship between 25[OH]D and depression and anxiety in pregnancy.

Thus, new, appropriate evidence is needed to meet the global need for a detailed and precise assessment of this association, indicating not only the results of evaluations of objective estimates, but also the methodological gaps identified in scientific practice, increasing transparency in science and guiding future research approaches. Therefore, this study systematically reviewed the literature to explore whether serum 25[OH]D concentrations/status are associated with the experience and/or severity of depressive or anxiety symptoms in women in the ante- and postnatal periods. An evaluation of the bibliometric indicators of the included studies is also described in this review.

## 2. Methods

### 2.1. Study Design and Open Science Practices

This systematic review and meta-analysis were based on the guidelines of the Cochrane Handbook for Systematic Reviews of Interventions [34] and the Preferred Reporting Items for Systematic Reviews and Meta-analyses (PRISMA) 2020. The PRISMA 2020 guidelines for abstracts were also checked [35].

This study adhered to the main practices of transparency in research and open science. This study is available in open access format. The protocol of this systematic review was hosted on PROSPERO [36] (CRD42023390895). All data related to this study are available at the Open Science Framework platform [https://osf.io/ukc9e/, accessed on 13 August 2024; doi:10.17605/OSF.IO/UKC9]. The authors declare that no artificial intelligence resources have been used to report the information described in this study—the content is original and unpublished.

### 2.2. Review Question and Search Strategy

This study was designed to gather evidence that could answer the following question: “Are serum concentrations or status of 25[OH]D associated with the presence and/or severity of anxiety and depression in adult women in the ante- and postnatal period?”.

The search keys, composed of MeSH terms/medical descriptors and Boolean operators, were organized to identify studies of interest in the PubMed database. The “PECO” strategy was used [37], which included related terms with (P)opulation: women aged at least 18 years during the ante- and postnatal periods; (E)xposure: insufficient and/or deficient serum concentrations of 25[OH]D; (C)omparison: adequate serum concentrations of 25[OH]D; and (O)utcome: experience and/or severity of anxiety or depression.

Five electronic bibliographic databases, including the PubMed, Scopus, Embase, Cochrane Library, and Web of Science databases, were screened on 30 September 2024. The full search strategies for the databases are available in Supplementary File S1. A manual search of the issues published in the last 10 years in the main journals on the following themes was conducted: *European Journal of Obstetrics and Gynecology and Reproductive Biology*; *Clinical Obstetrics and Gynecology*; and *The Journal of Maternal-Fetal & Neonatal Medicine*. The search for gray literature was also performed in the Google Scholar database via an adapted search strategy; only the first 300 studies were screened for eligibility. The list of references of all the studies after selection by full-text reading was screened to identify potential records not included in the preliminary search of the databases. No limits to the publication date or original language of publication were applied.

The results of the literature searches were uploaded to EndnoteWeb (https://web.endnote.com/, accessed on 30 September 2024; Thomson Reuters, New York, NY, USA), and duplicate records were automatically removed. Two researchers (MSF and LOLC) screened all titles and abstracts considering the selection criteria with Rayyan, a website for systematic reviews (https://www.rayyan.ai/, accessed on 30 September 2024) [38]. Hand searches were also performed by the same reviewers. The researchers independently evaluated the full texts of the included studies, and discrepancies were resolved by discussion with a third reviewer, an expert in epidemiology (AMA). The kappa agreement between researchers was calculated.

### 2.3. Eligibility Criteria

This systematic review included only cohort, case–control, and cross-sectional studies that assessed serum 25[OH]D concentrations in women (≥18 years of age) during pregnancy (antenatal or postnatal) and studies that used recognized and valid instruments/methods to explore the experience and/or severity of depressive and anxiety symptoms. Initially, it was erroneously decided to include studies of postnatal follow-up of women only up to a maximum of three months. However, considering that the protocols for the detection of postnatal symptoms are not universally consolidated, in addition to the fact that these mental symptoms (depressive and anxiety symptoms) can emerge later in motherhood, it was reasonable to include studies that investigated these outcomes within 1 year after childbirth, according to the Diagnostic and Statistical Manual of Mental Disorders (DSM-5) [39].

Review articles, letters to the editor, case reports, and case series were not considered. Based on previous studies, women with systemic and autoimmune diseases are more likely to suffer from depression and alterations in vitamin D metabolism during pregnancy and after childbirth [40,41]. Therefore, it was established that studies with samples of pregnant women with systemic/autoimmune diseases (e.g., multiple sclerosis, arthritis, diabetes, hypertension, asthma, and schizophrenia) would be excluded.

### 2.4. Data Extraction and Evidence Synthesis

A data extraction spreadsheet, which is based on the Cochrane Consumers and Communication Review Group’s data extraction template, was adapted for this study with Microsoft Excel [34]. Two reviewers (MSF and LOLC) extracted the following data from all included studies: year; country; journal name/impact factor (https://clarivate.com/first-time-journal-citation-reports-inclusion-list-2023/, accessed on 22 May 2024); study design; objective; sample (*n* per group); chronic diseases; season of the year; additional 25[OH]D supplementation; 25[OH]D measurement method (serum concentration); pregnancy period; adverse pregnancy outcomes; diagnostic criteria for anxiety; diagnostic criteria for depression; serum concentrations of 25[OH]D; main results—experimental group [mean and SD/*n* (%)]; main results—control group [mean and SD/*n* (%)]; main results—adjusted analysis; main conclusion; conflict of interest; financial support; open science practices (open access, protocol index; data sharing—www.force11.org/group/fairgroup/fairprinciples, accessed on 22 May 2024); AI statement; ethical approval; adequate use of guideline reports (e.g., https://www.equator-network.org/, accessed on 22 May 2024); and CRediT Author Statement. For the purpose of standardization, for all studies that presented the results of serum 25[OH]D assessment in nmol/L, the values were converted to ng/mL [42].

A third reviewer (AMA) double-checked all the extracted data. In the case of any missing data, the corresponding authors of the included studies were contacted via e-mail to provide additional data to be included in the quantitative analysis. A total of 25 authors were contacted. Authors who did not reply to requests were contacted one more time after two weeks; five authors ultimately provided the requested data (response rate: 20%) [43,44,45,46,47].

The following bibliometric aspects of the studies were explored [48]: journal name, impact factor, declaration of conflicts of interest and financial support, use of reporting guidelines, declaration of contributions by each author, ethical approval, and practice of transparency in science (i.e., previous publication of the study’s research protocol and information concerning open access or via request of the study’s sample database). Since many artificial intelligence (AI) textual writing resources have become available since 2020, studies published in the last four years (2020–2024) that present authors’ generative AI and AI-assisted technology statements were identified. Only one independent researcher extracted this information (MSF).

### 2.5. Risk of Bias Analysis and Certainty of Evidence

The Joanna Briggs Institute’s Critical Appraisal Checklist for cross-sectional studies (8 items) was used to identify the risk of bias of the included records [49]. This tool was used to generate responses by two independent reviewers (MSF and LOLC), namely “yes”, “no”, “unclear”, or “not applicable”. For each study, the total number of “yes” answers was considered in the elements of the checklist, and the study was classified as having a “low” risk of bias when the study achieved 70% or more “yes” answers, “moderate” if the proportion of “yes” answers was between 50% and 69%, and “high” risk of bias if the proportion of “yes” answers reached 49%.

Both independent reviewers (MSF and LOLC) appraised the quality assessment of cohort and case series studies via the Newcastle–Ottawa Scale (N-OS) [50]. The N-OS contains eight items categorized into three dimensions: selection, comparability, and outcome. A star scoring system was used for semiquantitative assessment of study quality. Studies are graded one star each for all items except comparability, which has the potential to score up to two stars, with the maximum possible score being nine. Studies are rated from 0–9, with those studies rated 0–2 (poor quality), 3–5 (fair quality), and 6–9 (good/high quality).

Disagreements between the reviewers in this step were resolved by discussion with a third reviewer (AMA). Kappa agreement between the examiners was determined.

The Grading of Recommendations Assessment Development and Evaluation (GRADE) tool (www.gradepro.org/) [51] was used to transpose the risk of bias into certainty of evidence. This assessment was performed by one researcher (FWMGM). The inconsistency, indirectness, imprecision, and other methodological and sampling aspects of the studies were considered to determine the certainty of the evidence.

### 2.6. Meta-Analysis

Review Manager software version 5.4 (Copenhagen: The Nordic Cochrane Centre, The Cochrane Collaboration) was used for the meta-analysis (quantitative analysis). Independent meta-analyses were performed for antenatal and postnatal depression. For postnatal depression meta-analyses, different analyses were pooled when 25[OH]Ds were collected during pregnancy or after delivery. The means of serum 25[OH]D were compared between women with and without depression using the mean difference (MD). Its 95% confidence interval (95% CI95% CI) was also estimated. For these analyses, subgroups were created according to the questionnaire used to assess depression.

For categorical variables, the presence of depression among women with or without deficiency [≤20 ng/mL] or insufficient [<30 ng/mL] 25[OH]D concentrations was also extracted to pool the odds ratio (OR) and its 95% confidence interval. Finally, studies that reported the adjusted OR (for at least two other covariables) for the association between depression and deficiency of 25[OH]D were also meta-analyzed. Therefore, eight meta-analyses were performed in the current study, all of which used a random effects model. Heterogeneity was assessed via the Q test and quantified via the I^2^ statistic. Statistical significance was established as *p* < 0.05. Due to the low number of studies included in each meta-analysis, no other analyses, such as sensitivity analysis, publication bias analysis, or meta-regression, could be performed.

Due to the high heterogeneity of the diagnostic methods used in these investigations and in the reporting of information, it was not possible to conduct any meta-analysis for the association between 25[OH]D concentrations and anxiety.

## 3. Results

### 3.1. Study Selection Process

**Figure 1:**
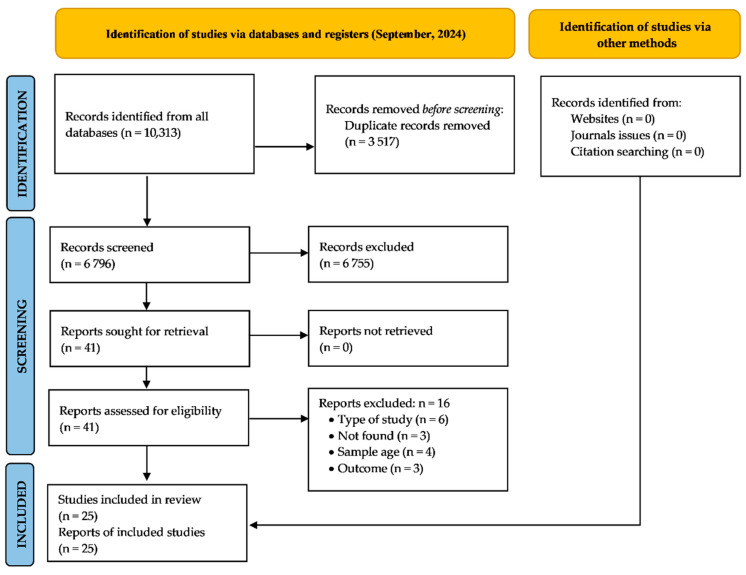
PRISMA 2020 flow diagram for searches of databases, registers and other sources.

The searches performed in all the electronic databases yielded 10,313 potentially relevant records [(PubMed, *n* = 2553), (Scopus, *n* = 2468), (Embase, *n* = 2183), (Cochrane Library, *n* = 1385), (Web of Science, *n* = 1724)]. The manual search strategy for the issues of journals and gray literature did not identify any additional records for inclusion. A total of 3517 duplicates were removed, and a total of 6796 records were subsequently screened by title/abstract reading. From these, 41 full texts were read, of which 16 were excluded and 25 were included [30,31,44,45,46,47,52,53,54,55,56,57,58,59,60,61,62,63,64,65,66,67]. The reasons for exclusion and the process of selecting the studies are detailed in Figure 1. The kappa coefficients between the two researchers were 0.75 and 0.99 for the title/abstract and full-text selection steps, respectively (i.e., substantial/satisfactory standard of agreement).

### 3.2. Risk of Bias of Included Studies

The analysis of the risk of bias of the cross-sectional studies [30,31,43,45,46,56,61,64,67] revealed that none presented a high risk of bias; more than half of the registries (*n* = 6) were classified as having a low risk of bias [31,43,45,56,61,64], whereas three studies were classified as having a moderate risk of bias [30,46,67] (Table 1).

Among the 15 cohort studies [32,33,44,47,53,54,55,57,58,59,60,62,63,65,66], 5 were classified as having a low risk of bias [47,54,55,57,66], 7 as having a moderate risk of bias [32,33,44,58,59,60,65], and 3 as having a high risk of bias [53,62,63]. The case-control studies were classified as having a low risk of bias [52] (Table 2). The main limitations present in all studies were related to the comparability of samples on the basis of design or statistical analysis. The kappa coefficients between the two researchers were 0.84, 0.79, and 0.100 for the cross-sectional, cohort, and case-control studies, respectively (i.e., substantial/satisfactory standard of agreement).

### 3.3. Methodological Aspects of the Included Studies

The general methodological descriptions of the studies included can be found in Table 3. A total of 15 registries were cohort studies [32,33,44,47,53,54,55,57,58,59,60,62,63,65,66], 9 were cross-sectional studies [30,31,43,45,46,56,61,64,67], and only 1 was a case-control study [52]. More than half of the studies were published between 2014 and 2024 [30,31,32,33,43,44,45,46,47,52,53,54,56,57,58,59,60,61,63,64,65,66,67]. Studies were conducted in different countries, namely the United States of America [31,33,53,54,56,59,60,62], India [45,64], China [57,61,67], Turkey [30,44,46], Australia [58,65], Brazil [47], the Netherlands [32,55], Iran [52], Iraq [63], Singapore [66], and Kuwait [43]. Twenty-four studies investigated the associations between depressive symptoms and serum 25[OH]D concentrations [30,31,32,43,44,45,46,47,52,53,54,55,56,57,58,59,60,61,62,63,64,65,66,67]. A total of four registries have explored the relationship between anxiety and 25[OH]D status in the prenatal period [30,31,32,33].

The studies used valid methods to identify the experience of depressive symptoms in the samples. The main adopted screening tools for depression were the Edinburgh Postnatal Depression Scale [43,44,45,46,47,54,57,58,59,60,61,62,63,64,65,66,67]; the Beck Depression Inventory [30,52,53]; the Center for Epidemiological Studies Depression [32,53,55,56]; and the Depression, Anxiety, and Stress Scale [31]. However, the studies that explored anxiety symptoms also used well-known screening indices (e.g., the Beck Anxiety Inventory [30]; the Depression, Anxiety, and Stress Scale [31]; the Perinatal Anxiety Screening Scale [33]; and the State–Trait Anxiety Inventory [32]).

The different tools used the predefined cutoff points of the total score; cutoff points were based on the mean/median of the total score and the global sum of all the responses obtained in the evaluation to categorize the experience (i.e., absence vs. presence), the symptom degree (i.e., no symptoms vs. mild symptoms vs. moderate symptoms vs. severe symptoms), and the global level (i.e., higher overall scores predict greater experience of the outcome) of depressive and anxiety symptoms. The serum concentration of 25[OH]D in women was measured via chemiluminescence [30,43,46,54,56,57,61,63], enzyme-linked immunosorbent assay (ELISA) [32,33,44,52,55,64,65], rapid direct radioimmunoassay [59,62], and liquid chromatography-tandem mass spectrometry analysis [31,47,53,58,60,66,67]. A variation in the reported serum concentration of 25[OH]D between studies was observed: (1) description on the basis of percentile distribution, (2) total serum concentration, and (3) endocrinological status of 25[OH]D (not sufficient vs. insufficient; not deficient vs. deficient).

A total of 21 studies used robust statistical analysis with adjusted models for confounding factors to verify the associations between serum 25[OH]D indicators and depressive [31,32,43,44,47,52,53,54,55,56,57,58,59,60,61,62,64,65,66,67] and/or anxiety [31,33] symptoms. The potential confounding factors considered included sociodemographic (e.g., ethnicity, age, and level of education) and economic (e.g., family income and employment status) characteristics, aspects of maternal health (e.g., body mass index, previous psychiatric treatment, use of medication, and vitamin or mineral supplementation), lifestyle habits (e.g., smoking, alcohol consumption, and physical activity), and factors related to pregnancy and parenthood (e.g., partner support and planned vs. unplanned pregnancy). Four studies conducted only bivariate analyses (e.g., chi-square test or Pearson’s ρ) [30,45,46,63].

### 3.4. Qualitative Synthesis of the Evidence

Different collection periods for the outcome (depression/anxiety) and main exposure (25[OH]D concentrations /status) indicators were adopted in the studies: (1) measurement of serum 25[OH]D and screening for depressive symptoms in the postnatal period [46,52,53,57,61,62,64]; (2) measurement of serum 25[OH]D in the antenatal period and screening for depressive symptoms in the postnatal period [32,44,54,58,65]; (3) longitudinal measurement of serum 25[OH]D and screening for depressive symptoms in both periods [59,60,63,66,67]; (4) measurement of serum 25[OH]D and screening for depressive symptoms in the antenatal period [30,31,43,45,47,55,56]; and (5) measurement of serum 25[OH]D and screening for anxiety symptoms in the ante- and postnatal periods [30,31,32,33]. Thus, a total of 12 studies explored the presence of depressive symptoms in the prenatal period [30,31,43,45,47,55,56,59,60,63,66,67], and 17 explored the presence of depressive symptoms in the postnatal period [32,44,46,52,53,54,57,58,59,60,61,62,63,64,65,66,67]. Three studies that screened for the presence of anxiety symptoms were conducted before delivery [30,31,33], and two studies explored the presence of anxiety in the postnatal period [32,33] (Table 4).

The prevalence rates of maternal depression and anxiety ranged from 7.1 to 49% and 12.4 to 37.8%, respectively. The prevalence rate of deficient/insufficient 25[OH]D status was 15.7–82.6%. Regardless of the type of statistical analysis, seven studies reported statistically significant associations between exposure (25[OH]D level/status) and both outcomes (depression (*n* = 7) [30,31,45,47,55,56,60]; anxiety (*n* = 1) [30]) in the prenatal period; other studies did not detect significant direct or indirect associations between these variables [31,32,33,43,59,63,66,67]. Among the studies that explored the presence of depressive symptoms in the postnatal period, nine detected significant associations between 25[OH]D concentrations/status and the prevalence or severity of depressive symptoms [44,52,53,57,60,62,64,65,67].

Six cohort studies analyzed the level/status of 25[OH]D and depressive symptoms in the prenatal period [47,55,59,60,63,66], three of which identified a statistically significant association [47,55,60]. Among the studies that considered the postnatal period, five reported a significant association between depression and 25[OH]D [44,53,60,62,65]. In addition, two cohort studies that screened for the presence of anxiety symptoms did not identify an association between ante- or postnatal anxiety and 25[OH]D [32,33].

Of the total of six records, four cross-sectional studies that included an antenatal assessment identified significant associations between 25[OH]D and depression [30,31,45,56], and half of those with postnatal assessment (*n* = 2) detected no association [46,61]. Finally, one cross-sectional study that screened for anxiety symptoms identified a significant association between anxiety levels and 25[OH]D deficiency [30].

Only the case-control study included in this review revealed an association between 25[OH]D deficiency and increased severity of depressive symptoms in women in the postnatal period [52].

### 3.5. Quantitative Synthesis of Evidence

**Figure 2:**
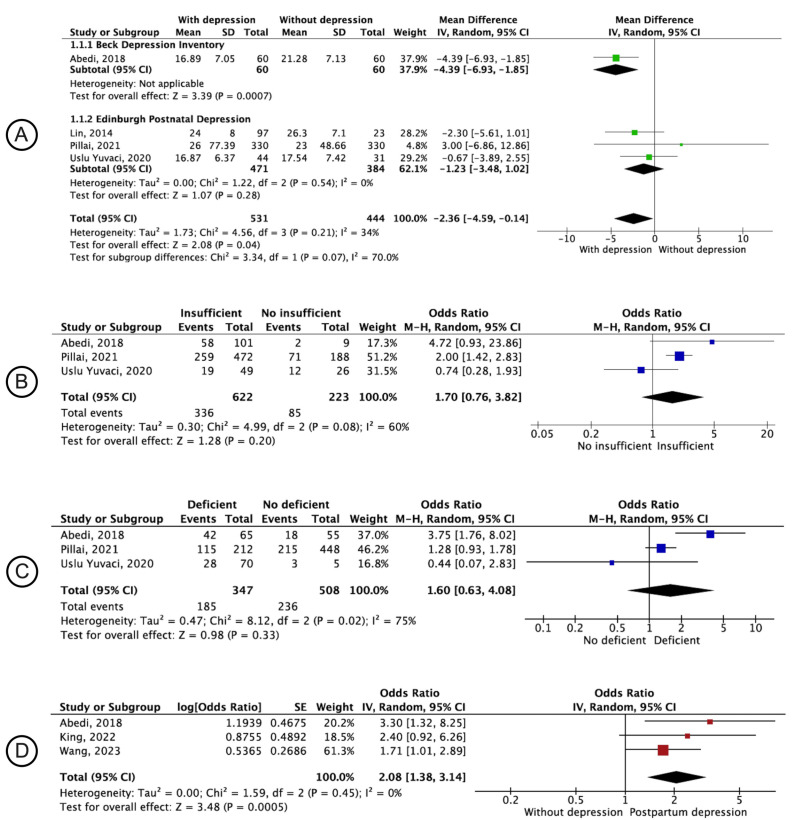
Forest plot of the pooled effect of the association between depression and vitamin D. (**A**), Postpartum depression outcome with 25[OH]D concentration measurement in the postnatal period. (**B**), Postpartum depression outcome and 25[OH]D concentration < 30 ng/mL natal. (**C**), Postpartum depression outcome and postnatal 25[OH]D concentration ≤ 20 ng/mL. (**D**), Postpartum depression outcome, with vitamin D deficiency ≤ 20 ng/mL measured postnatal (using only adjusted model estimates) [46,52,59,61,64,67].

Figure 2A shows the comparison of 25[OH]D concentrations measured in the postnatal period for postnatal depression. Postnatal 25[OH]D concentrations were shown to be, on average, 2.36 ng/mL (95% CI: −4.59; −0.14) lower in women with postnatal depression than in those without depression [46,52,61,64]. These findings are consistent with those of the Beck Depression Inventory Scale (MD: −4.39; 95% CI: −6.93; −1.85). However, no significant difference between the groups was observed for the Edinburgh Postnatal Depression Scale (MD: −1.23; 95% CI: −3.48; 1.02) [46,61,64]. Figure 2B shows the comparison of postnatal depression between women with and without insufficient 25[OH]D measured during the postnatal period. No significant difference was observed between the groups for this analysis (OR: 1.70; 95% CI: 0.76; 3.82). Figure 2C shows the comparison of postnatal depression in women with and without 25[OH]D deficiency measured postnatal; no significant association was identified (OR: 1.60; 95% CI: 0.63–4.08). The adjusted odds ratio for postnatal depression in relation to exposure to 25[OH]D deficiency measured in the postnatal period is shown in Figure 2D. In this analysis, only studies that performed adjusted analyses with at least two other exploratory variables for this association were included. Compared with those without 25[OH]D deficiency, women with 25[OH]D deficiency were 2.08-times more likely (95% CI: 1.38; 3.14) to have postpartum depression.

**Figure 3:**
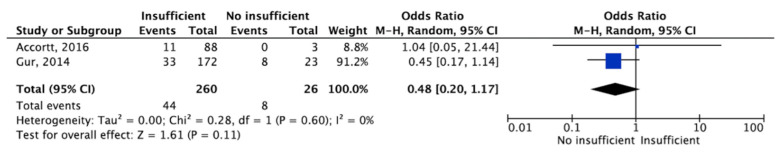
Postpartum depression in women with or without vitamin D insufficiency was measured in the antenatal period [44,54].

Figure 3 shows the comparison for postpartum depression in women with and without 25[OH]D insufficiency measured in the prenatal period. No significant associations were detected (OR: 0.48; 95% CI: 0.20; 1.17).

**Figure 4:**
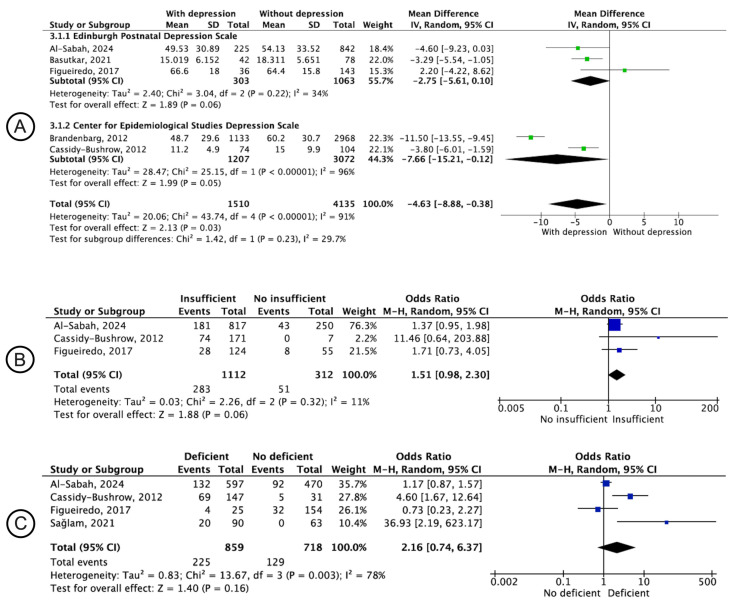
Forest plot of the pooled effect of the association between depression and vitamin D according to the period of clinical data collection and the instruments used. (**A**), 25[OH]D concentrations in the antenatal period and the prevalence of depression during pregnancy. (**B**), Depression during pregnancy in women with or without insufficient vitamin D measured during pregnancy. (**C**), Depression during pregnancy in women with or without deficiency of vitamin D measured during pregnancy [30,43,45,47,55,56].

Figure 4A presents the comparison of 25[OH]D concentrations measured in the antenatal period for depression during pregnancy. In the global analysis, significantly lower 25[OH]D concentrations were identified in women with depression (MD: −4.63; 95% CI: −8.88; −0.38) than in those without depression. In the subgroup of the Centre for Epidemiological Studies Depression scale, a significantly lower level of 25[OH]D was identified in women with depression (MD: −7.66; 95% CI: −15.21; −0.12). However, for the Edinburgh Postnatal Depression Scale, no difference between the groups was identified (MD: −2.75; 95% CI: −5.61; 0.10). Figure 4B shows the comparison of depression during pregnancy between women with and without insufficient 25[OH]D measured during pregnancy. Figure 4C shows the comparison of depression during pregnancy between women with and without 25[OH]D deficiency. For both analyses, no significant associations were identified (OR: 1.51; 95% CI: 0.98–2.30 and OR: 2.16; 95% CI: 0.74–6.37, respectively).

No estimated effect analysis was performed to verify the association between concentrations of 25[OH]D and anxiety.

### 3.6. Certainty of the Evidence

Supplementary File S2 [S2(A) and S2(B)] shows the GRADE assessment for each meta-analysis performed in the present study. A very low and low certainty of evidence was detected in the analyses between the global serum level (continuous variable) and the status (deficient/insufficient—dichotomous variable) of 25[OH]D in the maternal blood in ante- and postnatal depression. The reasons for downgrading were mainly study limitations due to a serious risk of bias (moderate and high risk), very serious inconsistency (high heterogeneity of analyses), and imprecision (absence of statistical significance between groups and very low number of individuals in groups) among the included studies.

### 3.7. Bibliometric Data

More than half of the studies (*n* = 19) were published in journals with impact factors (IFs) ≥ 2 (e.g., *Journal of Women’s Health*, *Nutrients Archives of Medical Research*, and *Archives of Women’s Mental Health*) [31,32,33,44,47,53,54,55,56,57,59,60,61,62,63,64,65,66,67]; three studies were published in journals with no IFs [30,45,52]; and the other three studies were published in journals with IFs < 2 [43,46,58]. All studies were performed with the approval of the Research Ethics Committee. The absence of direct or indirect conflicts of interest involving associations with commercial entities that supported the studies, associations with commercial agencies that may have an interest in publication of research results, and financial associations involving family members and other relevant nonfinancial associations was reported in 21 studies included in this review [30,31,32,43,45,46,52,53,54,55,57,58,59,60,61,62,63,64,65,67]. Four studies did not provide a conflict-of-interest statement [44,47,56,66]. The financial support received from research agencies and/or educational institutions was reported in 21 studies [30,31,32,33,43,46,53,54,55,56,57,58,59,60,61,62,63,64,65,66,67]. Five studies did not report any data related to research funding [44,45,47,52]. The statement of the use of generative AI/AI-assisted technologies in scientific writing was not found in any of the 14 studies included in this review that were published in the last quadrant (2019–2023).

The inclusion of the CRediT (Contributor Roles Taxonomy) Author Statement was found in 13/25 studies [30,32,43,46,53,54,57,58,59,61,63,66,67]. No study has described the use of reporting guidelines. No records reported anything about the prior indexing of the research protocol. Four studies published in 2022 reported information on access to the research database [32,43,59,63] (Table 4).

## 4. Discussion

The results of this systematic review with meta-analysis revealed a significant relationship between the serum concentration of 25[OH]D and ante- and postnatal depression. The estimated effect analysis revealed a statistically significant association between 25[OH]D status/level and the presence of antenatal depressive symptoms (MD: −4.63; 95% CI: −8.88; −0.38). Mothers who developed postpartum depression had lower 25[OH]D concentrations than those who did not experience depression (MD: −2.36; 95% CI: −4.59; −0.15). A significant association was identified between serum 25[OH]D and antenatal anxiety symptoms observed in the qualitative analysis in one study included.

Clinical studies performed with a general population sample have demonstrated that depression, anxiety, and cognitive impairment are associated with low concentrations of serum 25[OH]D [29,68]. Some plausible biological methods that could support this association include (A) increased region-specific expression of 25[OH]D receptors in brain areas (such as the prefrontal and cingulate cortices), which are known to play a key role in mood regulation; (B) the modulatory role of 25[OH]D in the association between depression/anxiety and inflammation; and (C) emerging insights into the neuroprotective properties of 25[OH]D (anti-inflammatory and antioxidant effects) [23,69,70]. This hormone binds to receptors in various brain regions, including the hippocampus and cingulate cortex, which are involved in the pathogenesis of common mental disorders [6]. Thus, it is noteworthy that substantial attention should be given to individuals with reduced concentrations of 25[OH]D, such as pregnant women, as these may have more severe systemic and mental effects [6]. Other negative maternal health outcomes have been linked with lower serum 25[OH]D concentrations (e.g., risk of premature birth, small babies for gestational age/low birth weight, recurrent miscarriage, bacterial vaginosis, and gestational diabetes mellitus) [71]. Clinical trials have already shown that a prenatal 25[OH]D screening and treatment program can be an effective approach to detecting deficient women, improving 25(OH)D concentrations and reducing adverse pregnancy outcomes [68,72].

Due to the small sample size, cross-sectional design, and lack of adjustment for confounding factors, the available evidence on maternal 25[OH]D concentrations during the pregnancy-puerperal cycle can be considered inconsistent [73]. In the medical community, 25[OH]D homeostasis during pregnancy is characterized by different stages of adaptation, namely (a) increased maternal calcitriol, (b) maternal availability of 25[OH]D to the neonate, and c) increased maternal concentrations of 25[OH]D-binding protein [8,74]. A gradual decrease in 25[OH]D serum concentrations over the gestational trimester has also been reported in previous studies [74,75]. During the gestational period (first three weeks), 25[OH]D concentrations in maternal blood may increase. However, longitudinal studies have reported that, due to the transfer of maternal 25[OH]D via the placental barrier to the primary reservoir of fetal 25[OH]D, a progressive decrease in total concentrations of 25[OH]D in maternal serum can also be observed from the first trimester of pregnancy [75,76].

The pathogenesis between 25[OH]D and depression/anxiety in the ante- and postnatal periods is complex and involves the neuromodulator role of the HPA axis, which reduces cortisol levels and increases the presence of 25[OH]D receptors and the enzyme 1-alpha-hydroxylase in the blood [77]. Calcitriol, when bound to 25[OH]D receptors on immune cells, plays a role in reducing the levels of inflammatory cytokines such as IL-6 and TNF-α. These cytokines, when present in high concentrations in the blood, influence the experience of depression and anxiety via disturbances in the HPA mechanism, stimulating an increase in cortisol levels [78]. Another function of calcitriol is related to the regulation of serotonin synthesis, increasing calcium metabolism, which acts as a second messenger for the rhythmic release of gonadotropin-releasing hormone by the hypothalamus. This hormone stimulates the production of follicle-stimulating hormone and luteinizing hormone by the pituitary gland, which in turn stimulates the ovaries to produce estriol, contributing to the increase in serotonin associated with the disruption of mood control [17]. Other studies have shown that serum concentrations of 25[OH]D are related to the presence of sleep disorders, suicide risk, and the development of neurodegenerative diseases during or after pregnancy [11,79].

This multidirectional relationship may also support the results of this study, which showed that a lower serum 25[OH]D concentration is significantly related to the prevalence of postpartum depression. This evidence was also identified in a systematic review with a meta-analysis that revealed a greater risk effect for postpartum depression among mothers with 25[OH]D deficiency (OR: 3.67; 95% CI: 1.72–7.85) than among those with 25[OH]D insufficiency [10]. Nevertheless, the pathophysiology of 25[OH]D and the occurrence of mental symptoms also justify another finding of this study, similar to that reported in another systematic review [11], which demonstrated that ante- and postnatal anxiety can be significantly associated with lower serum concentrations of 25[OH]D. Perinatal anxiety frequently cooccurs with perinatal depression [80].

Although a general association was identified between postnatal depression and 25[OH]D concentrations, there were differences in the instruments used to detect mental symptoms. Postnatal 25[OH]D concentrations were significantly lower in women with postnatal depression, as determined via the Beck Depression Inventory. However, there was no association between 25[OH]D concentrations and postnatal depressive symptoms in studies that used the Edinburgh Postnatal Depression Scale. The possible reason for this difference is that the Beck Depression Inventory has a greater number of somatic (physical) items than the Edinburgh Postnatal Depression Scale [81]. Indeed, several instruments are available for screening postpartum depressive symptoms, and on the basis of solid evidence published in the last decade, it is suggested that the Edinburgh Postnatal Depression Scale should be preconized for the diagnosis of postpartum depression in women over 18 years of age [82].

A previous systematic review and meta-analysis did not identify an association between serum 25[OH]D concentrations and antenatal depression (OR: 1.47, 95% CI: 0.92–2.36) [10], which contrasts with the evidence found in this study. The influence of other proximal and distal factors during pregnancy on the presence of depressive symptoms in the women included in the previous study may act individually and synergistically [10]. It is also possible that the statistical insignificance is due to the presence of methodological limitations in the studies included in the previous review (e.g., small sample size, follow-up period, and statistical adjustment for mental health variables), which may underestimate or overestimate the direction of the associations found. Another important factor to consider in order to justify this finding is the heterogeneity of the periods during which 25[OH]D was collected in the included studies, taking into account the homeostatic level of serum.

The bibliometric analysis conducted in this research revealed that most of the records included were published in journals of notable relevance in medical science. More than two-thirds of the included studies provided information on conflicts of interest and funding sources. Overall, although a conflict of interest should not necessarily have a negative impact on research results, providing more information about the relationship between the author and funding sources allows the reader to evaluate the literature more carefully [83,84]. Studies performed in other medical specialties have reported that research funded by the private sector is more likely to report positive results [85], which was not identified among the studies in this review that received non-institutional funding. In addition, half of the records clearly presented the attributes of each author in the development and dissemination of the data, which can benefit the academic community, facilitating communication between researchers and recognizing the diverse skills and resources they bring to research groups [86].

No included study adopted the use of reporting guidelines to describe the information from their studies. Adherence to reporting guidelines, including explicit descriptions of any deficiencies in study design, can minimize bias, improve reproducibility, and facilitate the clear, transparent, and complete reporting of research findings [87]. Promoting transparency in the creation of scientific knowledge and increasing access to scientific production have been the main objectives of the open science movement. The most visible aspect of the open science movement has been open access to scientific studies and published results, while the availability of research protocols and data sharing are more infrequent practices [88]. Due to Brazil’s open access funding policies, all studies were easily accessible via institutional platforms, but nearly half (11/25) of these studies were published in subscription-based platforms and are not widely available to the scientific community or society. No study supplied direct access to the research protocol, which could provide researchers with a broader perspective on research logistics, the instruments used, and the approaches adopted in the management and analysis of the data. The reporting of information about data sharing was identified in only four studies, and none of these studies provided access to the database via institutional repositories or similar platforms. In the execution of this review, the authors of all studies were contacted via email to provide data that could be included in the estimated effect analysis. Unfortunately, a low response rate was obtained, and few studies were included in the meta-analysis. Thus, future studies must incorporate open science practices throughout their entire development to ensure that their research and data can be interpreted and verified/reanalyzed to test new hypotheses.

The limitations of this study should be highlighted and considered when the findings are interpreted. Few data were included in the meta-analysis due to the lack of information and the different approaches used in screening and defining depressive and anxiety symptoms. Furthermore, most studies did not report information on the history of 25[OH]D supplementation or dietary intake of 25[OH]D-rich foods, which may suggest a potential risk of bias. Most studies assessed postnatal depression in a short period, and more than half of them used self-administered questionnaires rather than structured interviews. The small sample size of most studies is a limitation since the sample size should be larger to increase the power to detect associations. The lack of unpublished studies may limit the findings, especially regarding anxiety symptoms.

The strengths of this study should also be listed. This systematic review with meta-analysis performed standardized analyses considering the period of collection of information related to mental symptoms and serum 25[OH]D measurement. For this study, a vast literature screening was used to update the results of previous reviews that used similar analysis methodologies. The two most prevalent mental disorders in the pregnancy–puerperal period was investigated, expanding the understanding of the influence of 25[OH]D on the occurrence of depressive and anxiety symptoms. This is the first systematic review on the subject that analyzed the bibliometric aspects of the included records, emphasizing the impact of transparency and data-sharing practices in medical science.

## 5. Conclusions

Through this systematic review, it was confirmed that ante- and postpartum depression are associated with a reduced serum of 25[OH]D concentrations. Another important point is that anxiety symptoms in the antenatal period may not be associated with a low concentration of 25[OH]D. The clinical importance of the results of this study is remarkable since solid evidence of the association between serum 25[OH]D concentrations allows for the formulation of practices and actions aimed at preventing maternal and fetal complications, identifying risk factors, and promoting early interventions, such as vitamin D supplementation. This approach could not only improve the mental and physical health of pregnant women by reducing the risk of depression and its adverse effects but also reduce healthcare costs by avoiding more complex treatments. In addition, understanding this relationship could guide public health guidelines and awareness campaigns, comprehensively benefiting mothers and babies.

## Figures and Tables

**Table 1:** Assessment of the risk of bias in cross-sectional studies (*n* = 9).

Author (Year)	JBI for Analytical Cross-Sectional Studies								Total Score
	Q1	Q2	Q3	Q4	Q5	Q6	Q7	Q8	
Al-Sabah et al., 2024 [43]	Yes	Yes	Yes	Yes	Yes	Yes	Yes	Yes	8/8—Low RoB
Basutkar et al., 2021 [45]	Yes	Yes	Yes	Yes	Yes	Yes	Yes	Yes	8/8—Low RoB
Cassidy-Bushrow et al., 2012 [56]	Yes	Yes	Yes	Yes	Yes	Yes	Yes	Yes	8/8—Low RoB
Huang et al., 2014 [31]	Yes	Yes	Yes	Yes	Yes	Yes	Yes	Yes	8/8—Low RoB
Lin et al., 2019 [61]	Yes	Yes	Yes	Yes	Yes	Yes	Yes	Yes	8/8—Low RoB
Pillai et al., 2021 [64]	Yes	Yes	Yes	Yes	Yes	Yes	Yes	Yes	8/8—Low RoB
Sağlam et al., 2021 [30]	Yes	Yes	Yes	Yes	No	No	Yes	Yes	6/8—Moderate RoB
Yuvaci et al., 2020 [46]	Yes	Yes	Yes	Yes	No	Yes	Yes	Unclear	6/8—Moderate RoB
Wang et al., 2023 [67]	Yes	Yes	Yes	Unclear	Yes	Yes	Yes	Yes	7/8—Moderate RoB

Legend: Q1, Were the criteria for inclusion in the sample clearly defined? Q2, Were the study subjects and the setting described in detail? Q3, Was the exposure measured in a valid and reliable way? Q4, Were objective, standard criteria used for measurement of the condition? Q5, Were confounding factors identified? Q6, Were strategies to deal with confounding factors stated? Q7. Were the outcomes measured in a valid and reliable way? Q8,Was appropriate statistical analysis used?

**Table 2:** Risk of bias (RoB) analysis of cohort (*n* = 15) and case–control studies (*n* = 1).

Newcastle—Ottawa Quality Assessment Scale Cohort Studies	Selection				Comparability	Exposure			Definition of RoB
	(1) Representativeness of the Exposed Cohort	(2) Selection of the Non-Exposed Cohort	(3) Ascertainment of Exposure	(4) Demonstration That Outcome of Interest Was Not Present at Start of Study	(1) Comparability of Cohorts on the Basis of the Design or Analysis	(1) Assessment of Outcome	(2) Was Follow-Up Long Enough for Outcome to Occur	(3) Adequacy of Follow Up of Cohorts	
Accortt et al. (2021) [53]	★	-	★	-	★	-	★	★	Poor
Accortt et al. (2016) [54]	★	★	★	★	★ ★	★	★	★	Good
Brandenbarg et al. (2012) [55]	★	★	★	★	★ ★	★	★	★	Good
Desirée et al. (2024) [32]	★	★	★	-	★	★	★	★	Fair
Figueiredo et al. (2017) [47]	★	★	★	★	★ ★	★	★	★	Good
Fu et al. (2015) [57]	★	★	★	★	★ ★	★	★	★	Good
Gould et al. (2015) [58]	★	★	★	★	★	★	★	★	Fair
Gur et al. (2014) [44]	★	★	★	-	★	★	★	★	Fair
Hannan et al. (2023) [33]	-	★	★	★	★	★	★	★	Fair
King et al. (2022) [59]	★	★	★	★	★	★	★	★	Fair
Lamb et al. (2018) [60]	★	★	★	★	★	★	★	★	Fair
Murphy et al. (2010) [62]	★	★	★	★	-	★	★	★	Poor
Nassr et al. (2022) [63]	★	★	★	★	-	★	★	★	Poor
Robinson et al. (2014) [65]	★	★	★	-	★	★	★	★	Fair
Van Lee et al. (2020) [66]	★	★	★	★	★ ★	★	★	★	Good
**Newcastle—Ottawa Quality Assessment Scale Case Control Studies**	**Selection**				**Comparability**	**Exposure**			
	**(1) Is the Case Definition Adequate?**	**(2) Representativeness of the Cases**	**(3) Selection of Controls**	**(4) Definition of Controls**	**(1) Comparability of Cases and Controls on the Basis of the Design or Analysis**	**(1) Ascertainment of Exposure**	**(2) Same Method of Ascertainment for Cases and Controls**	**(3) Non-Response Rate**	
Abedi et al. (2018) [52]	★	★	★	★	★	★	★	★	Good

Legend: The star (★) is the standard symbol of the NOS scale; each ★ signifying a point on the NOS scale.

**Table 3:** Methodological aspects and main results of the included studies (*n* = 25).

Author Year Country Study Design	Objective Sample (N)—Age	Depressive Symptoms Screening and Classification Instrument [30,31,32,43,44,45,46,47,52,53,54,55,56,57,58,59,60,61,62,63,64,65,66,67]	Vitamin D Aspects of Measurement and Classification	Main Results (Descriptive Results, Bivariate Analysis, and Adjusted Analysis)	Highlights
Abedi et al., 2018 [52] Iran Case–control study	To evaluate the relationship between 25[OH]D and PPD in Iranian women of reproductive age. Experimental group (*n* = 60); Control group (*n* = 60) Without PPD: 26.43 (4.27) years With PPD: 27.6 (4.73) years	BDI -PPD (NR)	ELISA method from immunodiagnostic System Limited. Deficiency: <10 ng/mL Moderate insufficiency: 10–20 ng/mL Mild insufficiency: 20–30 ng/mL Normal: >30 ng/mL Sufficient: <20 ng/mL Deficient: >20 ng/mL	Experimental group: 16.89 (7.05) Control group: 21.28 (7.13) *p* = 0.001 Level: experimental vs. control >30 ng/mL: 2 (3.3); 7 (11.7) 21–29 ng/mL: 16 (26.7); 30 (50) <20 ng/mL: 32 (53.3); 19 (31.7) <10 ng/mL: 10 (16.7); 4 (6.7) Adjusted analysis OR: 3.30 (1.32–8.24); *p* = 0.01 Adjusted for age, education, education of husband, economic situation, and body mass index.	Women with PPD had a lower mean of 25[OH]D. Also, the number of women with moderate insufficiency and severe deficiency was significantly higher in the PPD group than those with normal 25[OH]D concentrations.
Accortt et al., 2016 [54] United States of America Cohort study	To examine associations between prenatal 25[OH]D status and PPD in a sample of African American women. N = 91 26 (5.9) years	EPDS -PPD: EPDS ≥ 12	Competitive chemiluminescence immunoassay. Inadequacy/deficiency: ≤20 ng/mL Mild insufficiency: 20–30 ng/mL Normal: >30 ng/mL	Adjusted analysis: β = −0.209, *p* = 0.058 Adjusted for maternal age, education, marital status, history of depression, season of vitamin D measurement, and pre-pregnancy BMI.	Low prenatal concentrations of 25[OH]D were not associated with PPD symptoms in African American women.
Accortt et al., 2021 [53] United States of America Cohort study	To investigate the association between depressive symptoms and lower 25[OH]D status, measured by the 25[OH]D receptor, in pregnant women. N = 89 27.80 (5.87) years	BDI—AD Total BDI score -BDI ≥ 10: Mild symptoms -BDI ≥ 19: Moderate/severe symptoms CES-D—PPD -CES-D ≥ 16: Risk for PPD.	Liquid chromatography–tandem mass spectroscopy Deficient: 25(OH)D ≤ 20 ng/mL	Antenatal 25[OH]D (*p* = 0.093) No PPD risk: 20.63 (7.68) ng/mL PPD risk: 17.78 (7.00) ng/mL Antenatal 25[OH]D < 20 ng/mL (*p* = 0.66) Not at PPD risk: *n* = 30 (50.8) PPD risk: *n* = 17 (56.7) Pregnancy VMR (*p* = 0.003) Not at PPD risk: 0.1026 (0.023) ng PPD risk: *n* = 0.0876 (0.017) ng Adjusted analysis: 25[OH]D Metabolite ratio, body mass index, maternal age, smoking, race, and pre- and postnatal depression.	Lower concentrations of circulating 25[OH]D or 25[OH]D < 20 ng/mL were significantly associated with PPD risk.
Al-Sabah et al., 2024 [43] Kuwait Cross-sectional	To estimate the prevalence of depressive symptoms during pregnancy and investigate factors associated with this condition among participants in the Kuwait Birth Study. N = 1070 31.46 (5.28) years	EPDS -EPDS < 13: Without depressive symptoms -EPDS ≥ 13: With depressive symptoms	Competitive chemiluminescence immunoassay. Inadequacy/deficiency: ≤30 ng/mL Sufficient: >30 ng/mL	Antenatal depression Deficiency/insufficiency vs. Sufficiency: 0.73 [0.50–1.05]; *p* = 0.093. Adjusted for NR	25[OH]D status was not significantly associated with depressive symptoms during pregnancy.
Basutkar et al., 2021 [45] India Cohort study	To examine the prevalence of AD symptoms in 25[OH]D deficient pregnant women. Depressive (*n* = 42); non-depressive (*n* = 78) Depressive—23.62 (2.73) years Non-depressive 23.56 (2.43) years	EPDS -EPDS > 12: Major depression -EPDS < 12: Minor depression	NR s-25(OH)D Deficient < 30 ng/mL	Depressive vs. non-depressive (*p* = 0.011) Deficient: (*n* = 32) vs. (*n* = 40) Insufficient (*n* = 10) vs. (*n* = 38) 25[OH]D (ng/mL) (*p* = 0.004) Depressive: 15.019 ± 6.152 Non-depressive: 18.311 ± 5.651 EPDS score with s-25[OH]D (ng/mL): Pearson-correlation: −0.294 Adjusted: Beta = −0.258 (*p* = 0.003) Adjusted for maternal age, education, household income, occupation, diet, sun exposure, season of 25[OH]D measurement, and parity.	The low s-25(OH)D levels were significantly correlated with antenatal depressive symptoms.
Brandenbarg et al., 2012 [55] Netherlands Cohort study	To examine low maternal 25[OH]D status as a potential risk factor for high levels of depressive symptoms in a pregnant population. Depressive symptoms Low/Normal (G16)—*n* = 2968 (72.4%) High (Q16)—*n* = 1133 (27.6%) 31.0 (4.8) years	CES-D -CES-D > 16: High levels of depressive symptoms	ELISA method from immunodiagnostic System Limited. Deficient ≤ 12 ng/mL Insufficient: 12–20 ng/mL Sufficient: 20 ng/mL Normal: >32 ng/mL	Adjusted analysis Total sample—(Low vs. high depressive symptoms) -Deficient: OR = 1.48, 95% CI: 1.13–1.95 -Insufficient: OR = 1.44, 95% CI: 1.12–1.85 -Sufficient: OR = 1.21, 95% CI 0.97–1.51 Adjusted for age, ethnicity, educational level, household income, employment status, and parity.	Women with low early pregnancy 25[OH]D status have a higher risk of elevated depressive symptoms in pregnancy.
Desirée et al., 2024 [32] Netherlands Cohort study	To investigate whether deficient antepartum vitamin D status is a risk factor for increased PPD and/or anxiety symptoms. N = 2483 NR	CES-D—PPD -CES-D ≥ 16: With PPD. STAI—Anxiety -STAI > 42: With anxiety	ELISA method from immunodiagnostic System Limited. Deficient: ≤12 ng/mL Insufficient (12–20 ng/mL) Sufficient: (20–32 ng/mL) Normal: ≥32 ng/mL	Depression [B= −0.13, 95% CI (−0.01. 0.26), *p* = 0.063] Anxiety [B = 0.17, 95% CI (0.03, 0.30), *p* = 0.017] Adjusted for maternal age, maternal educational level, ethnicity, employment, marital status, body mass index, and parity.	25[OH]D deficiency during pregnancy was associated with increased postpartum anxiety symptoms but not with PPD symptoms.
Cassidy-Bushrow et al., 2012 [56] United States of America Cohort study	Examined if early pregnancy 25[OH]D nutrition was associated with AD symptoms among African American women in the second trimester of pregnancy. CESD < 16 *n* = 104, 58.4% CESD ≥16 *n* = 74, 41.6%” Without/With depressive symptoms: 26.9 (6.2) years/25.8 (5.2) years	CES-D -CES-D > 16: Depressive symptoms	Liquid chromatography–tandem mass spectroscopy Deficient: <12 ng/mL Inadequate: 12–20 ng/mL Sufficient: 20–30 ng/mL No additional value: 30–50 ng/mL Potentially toxic: ≥50 ng/mL	Adjusted analysis A significant inverse relationship was found between log (25-OHD) and CES-D > 16 (OR: 0.54, 95% CI: 0.29–0.99, *p* = 0.046). For every 1-unit increase in log (25-OHD) (corresponding to 2.72 ng/mL increase in 25-OHD), the OR of CES-D > 16 decreased by 46%. Adjusted for maternal age, education, and marital status, season of 25[OH]D measurement and time between 25[OH]D measurement, and CES-D measurement.	Low concentrations of 25[OH]D in early pregnancy are associated with AD symptoms.
Figueiredo et al., 2017 [47] Brazil Cohort study	To investigate the association between concentrations of 25[OH]D with the occurrence of depressive symptoms in pregnancy. Without depressive symptoms (*n* = 143) With depressive symptoms (*n* = 36) Total sample: 26.8 (5.6) years Without depressive symptoms: 26.7 (5.4) years With depressive symptoms: 27.4 (6.4) years	EPDS -EPDS < 13: Without depressive symptoms -EPDS ≥ 13: With depressive symptoms	Liquid chromatography–tandem mass spectrometry. Deficient: <20 ng/mL Sufficient 20–30 ng/mL Normal: <30 ng/mL	EPDS < 13 vs. EPDS ≥ 13 25[OH]D (nmol/L)—143 [66.6 (18.0)] vs. 36 [64.4 (15.8)] 25[OH]D Status: 25[OH]D < 75 nmol/L (*p* = 0.216)/25[OH]D < 50 nmol/L (*p* = 0.403)/ 25[OH]D < 30 nmol/L (*p* = 0.508) No: 47 (32.9)/ 122 (85.3)/ 140 (97.9) vs. 8 (22.2)/ 32 (88.9)/ 36 (100.0) Adjusted analysis First trimester 25[OH]D (nmol/L): OR = 0.98, 95% CI 0.96–0.99—*p* = 0.047 25[OH]D (nmol/L) throughout pregnancy: OR = 0.99, 95% CI 0.98–1.01, *p* = 0.982 Adjusted for age, body mass index, skin color, schooling, alcohol consumption, smoking, and previous history of depression.	A higher prevalence of 25[OH]D inadequacy and depressive symptoms during the first trimester was observed. Higher 25[OH]D concentrations in the first trimester were associated with a decrease of 2% in the odds for presenting depressive symptoms throughout pregnancy.
Fu et al., 2015 [57] China Cohort study	To assess the possible relationship between serum l concentrations of 25[OH]D collected 24 h after delivery and PPD in a Chinese cohort sample. *n* = 213 Median: 31 (IQR: 29–32) years	EPDS -EPDS ≥ 12: Postpartum depression	Electrochemiluminescence for immunoassay analysis. Sufficient: <20 ng/mL Deficient >20–30 ng/ml	25[OH]D—*p* < 0.0001, median (IQR) With no PPD: 14.3 (10.2–18.2) ng/mL With PPD: 8.3 (7.5–9.3) ng/mL Negative correlation between concentrations of 25[OH]D and the EDPS score (r = 0.293) *p* < 0.0001) Adjusted analysis PPD was associated with ≤10.2 ng/mL (OR: 7.17, 95% CI: 3.81–12.94; *p* < 0.0001) Adjusted for age, breastfeeding, stressful life events, maternal education, family income, partner support, planned versus unplanned pregnancy, mode of delivery, and previous psychiatric contact.	Serum 25[OH]D concentrations after delivery are negatively associated with PPD.
Gould et al., 2015 [58] Australia Cohort study	To determine the association between 25[OH]D at delivery and the subsequent risk of PPD at six weeks and six months postpartum. N = 1040 ≤24 years: *n* = 248; 25–29 years = 306; 30–34 years: 311; ≥35 years: 175.	EPDS -EPDS < 13: Without depressive symptoms -EPDS ≥ 13: With depressive symptoms	Liquid chromatography–tandem mass spectroscopy method. Use of Cord blood. <12 ng/mL 12–20 ng/mL >20 ng/mL	Six weeks (RR: 0.92 95% CI: 0.84–1.02, *p* = 0.11) Six months postpartum (RR: 0.96 95% CI 0.88–1.05, *p* = 0.41) Adjusted analysis (OR: 1.74, 95% CI: 0.89–3.42) Adjusted for maternal age, race, parity, body mass index, education, previous history of depression, supplement use during pregnancy, and smoking status.	No consistent association between cord blood 25[OH]D and PPD at six weeks and six months postpartum.
Gur et al., 2014 [44] Turkey Cohort study	To evaluate a possible association between PPD and serum concentrations of 25-hydroxy 25[OH]D3 (25 (OH)D3) during mid-pregnancy. Baseline (*n* = 208); 1 week (*n* = 189); 6 weeks (*n* = 184); 6 month (*n* = 179). 28.5 years	EPDS. -EPDS ≥ 12: With PPD	ELISA method from immunodiagnostic System Limited. Mild 25[OH]D deficiency: 10–20 ng/mL Severe 25[OH]D deficiency: <10 ng/mL	Negative correlations (Person correlation) of 25[OH]D level and EPDS scores by time-points: 1st week: r = −0.2 (*p* = 0.02); 6th week: r = −0.2 (*p* = 0.01); 48 weeks: r = −0.3 (*p* < 0.01). Adjusted analysis [OR: 8.21, 95%IC: 2.8–23.45] Adjusted for maternal age, body mass index, season, 25[OH]D supplementation, and parity.	Lower maternal 25[OH]D 3 concentrations in the second trimester of pregnancy were associated with higher levels of PPD symptoms at 1 week, 6 weeks, and 6 months postpartum.
Hannan et al., 2023 [33] United States of America Cohort study	To examine whether an association exists and compare the distribution of 25[OH]D concentrations in women with and without anxiety symptoms. N = 101 NR	PASS. -PASS ≥ 21: With anxiety	ELISA method from immunodiagnostic System Limited. Optimal: >20 ng/mL Sub-optimal: <20 ng/mL	Bivariate analysis There was no significant difference in 25[OH]D concentration by anxiety category (*p* = 0.972). Adjusted for: NR.	No statistical difference was found between women with sub-optimal or optimal serum 25[OH]D concentrations in both periods.
Huang et al., 2014 [31] United States of America Cohort study	Investigate associations of early pregnancy serum 25[OH]D concentrations with self-reported depressive and anxiety symptoms. 33.4 (4.2) years	DASS-21 -Depression subscale -Anxiety subscale	Liquid chromatography–tandem mass spectroscopy. Classified in quartiles of the serum total 25[OH]D distributions (quartiles based on distributions among all study participants).	Serum 25[OH]D concentrations (ng/mL) Depression subscale Total sample: 4.4 (5.1)–5.8% (29) Quartile 1: 4.3 (5.5)–4.0% (5) Quartile 2: 4.4 (4.8)–7.3% (9) Quartile 3: 3.7 (4.4)–4.0% (5) Quartile 4: 5.4 (5.4)–8.0% (10) Anxiety subscale Quartile 1: 3.5 (4.3)–12.0% (15) Quartile 2: 4.5 (4.4)–14.5% (18) Quartile 3: 4.5 (5.5)–10.5% (13) Quartile 4: 4.6 (4.3)–12.8% (16) Adjusted for: body mass index and income.	Lower concentrations of 25[OH]D are associated with increased depression/anxiety symptoms in antenatal period.
King et al., 2022 [59] United States of America Cohort study	To examine the relationship between sleep disturbances, anxiety symptoms and 25[OH]D deficiency as early as the first trimester of pregnancy and clinically significant depressive symptoms later in pregnancy or in the postpartum period. (N = 105) 27.8 (5.36) years	EPDS -EPDS < 10: without depressive symptoms -EPDS ≥ 10: with depressive symptoms	Radioimmunoassay Sufficient: >20 ng/mL Deficient: ≤20 ng/mL	Women with deficient concentrations of 25[OH]D (≤20 ng/mL) were more likely to experience an EPDS score of ≥10 at follow-up assessments, but findings were not statistically significant (OR: 2.40; 95% CI 0.92–6.27). Adjusted for age, race, income, education, and numbers of pregnancies.	25[OH]D disturbances in early pregnancy are associated with an increase in peripartum depression.
Lamb et al., 2018 [60] United States of America Cohort study	This study investigated the relationship between maternal and cord blood 25[OH]D and maternal depressive symptoms over the perinatal period. N = 125 Mean (SD) 33.2 (5.3) years	EPDS -EPDS > 15: with severe depressive symptoms -EPDS > 15: without severe depressive symptoms	Liquid chromatography–mass spectrometry. Deficiency: <20 ng/mL	EPDS scores at baseline were significantly correlated with 25OHD < 20 ng/mL. Women with severe depressive symptoms had significantly lower 25OHD concentrations (t = −2.09, *p* = 0.039). An inverse correlation between 25OHD concentrations and depressive symptoms were observed at all three time points. For women who completed all time points (*n* = 88), mean depressive symptoms for women with 25[OH]D deficiency (<20 ng/mL) (*n* = 11) were lower than women without (*n* = 77; t = −2.09, *p* = 0.039) Adjusted for family history of depression and maternal 25[OH]D supplementation.	Levels of depressive symptoms were associated with perinatal period.
Lin et al., 2019 [61] China Cross-sectional study	To investigate the association between nutritional status and postpartum depressive symptoms at 6–8 weeks postpartum. Non-PPDS (*n* = 97); PPDS (*n* = 23); N = 120 Non-PPDS [mean: 32.6 (4.5)]; PPDS [mean: 31.6 (4.3)]	EPDS -EPDS ≥ 10: PPD symptoms	Electro chemiluminescence Insufficiency: <30 ng/mL Deficiency: <20 ng/mL	HGB (g/dL)—*p* = 0.197—PPSS vs non-PPSS Mean—26.3 (7.1) vs. 24.0 (8.0); 25[OH]D insufficiency: 41% vs. 35% 25[OH]D deficiency: 26% vs. 48% Adjusted analysis: No significant association were observed between PPDS and serum 25[OH]D 3—β = 0.031; SE = 0.033, OR = 1.032; *p*-value = 0.352; 95% CI = 0.966–1.101. Adjusted for: type of postpartum confinement, overall psychological stress scores, and postpartum care satisfaction scores.	25[OH]D insufficiency and deficiency was high in both groups, and no statistical differences were identified between the groups.
Murphy et al., 2010 [62] United States of America Cohort study	To determine whether a relationship exists between symptoms associated with PPD and 25[OH]D concentrations and to determine if serum 25(OH) D concentrations can predict the incidence of symptoms associated with postpartum depression. N = 97 28.9 (5.5) years	EPDS NR	Direct radioimmunoassay 25[OH]D Insufficiency: ≤32 ng/mL 25[OH]D Sufficiency: >32 ng/mL	Adjusted mean EPDS sum scores Mothers with lower concentrations of 25[OH]D had higher EPDS sum scores over time than mothers with higher 25[OH]D concentrations [Estimate = 0.659; SE = 0.051; F-value: 168.6 *p* < 0.001]. Adjusted for age, gender, educational level, marital status, insurance level, season, breast-feeding or bottle feeding, 25[OH]D dosage, and whether or not the pregnancy was planned.	A significant relationship over time was found between high EPDS scores and low 25[OH]D concentrations during the first 7 months postpartum (*p* = 0.02). Therefore, if a pregnant or postpartum woman is identified with insufficient 25[OH]D concentrations, she may be more at risk for developing symptoms associated with postpartum depression.
Nassr et al., 2022 [63] Iraq Cohort Study	To assess perinatal depressive symptoms associations with inflammatory markers and 25[OH]D concentrations, and to investigate the interaction between 25[OH]D and the inflammatory markers. N = 80 27.0 years (5.6) years	EPDS -EPDS ≥ 13: probable depression.	Electro chemiluminescence Deficient: ≤20 ng/mL Insufficient: 21–29 ng/mL Sufficient: ≥30 ng/mL	There was no statistically significant relationship between the antenatal or postpartum symptoms EPDS (6 months) score and 25[OH]D concentrations. Spearman’s rho and *p*-value = (1) Antepartum EPDS score; (2) Postpartum EPDS score: (1) −0.05 and *p* = 0.648; (2) 0.10 and *p* = 0.460. Adjusted for NR.	Pregnancy depressive symptoms were not associated with 25[OH]D concentrations.
Pillai et al., 2021 [64] India Cross-sectional study	To assess the serum concentrations of total, free, and bioavailable 25-hydroxy25[OH]D (25[OH]D) concentrations in women with postpartum depressive symptoms (PPD) and the association between 25[OH]D concentrations and PPD at 6 week post-delivery. Cases (*n* = 330) and control (*n* = 330) Total sample: mean 26 (4) years EPDS < 10: 25 (23–28) EPDS ≥ 10: 25 (23–29)	EPDS -EPDS< 10: without depressive symptoms -EPDS ≥ 10: with depressive symptoms	ELISA competitive enzyme linked immunoassay Deficient: <20 ng/mL Insufficient: 20–30 ng/mL Sufficient: ≥30 ng/mL	A significant negative correlation was identified between serum 25[OH]D concentrations and EPDS score in total study subjects (*p* < 0.001, r = −0.19) Adjusted analysis 25[OH]D serum total (B = −0.19, t = −5.92) Free (B = −0.13, t = −3.85) Bioavailable 25[OH]D concentrations (B = −0.13, t = −3.86). Adjusted for age, body mass index, socioeconomic status of the mother, not happy with married life, presence of adverse life events during pregnancy, presence of excess fear of labor during delivery, presence of medical conditions during pregnancy, kangaroo mother care given, and presence of child care stressful for the mother.	The results indicate that significantly lower concentrations of total, free and bioavailable serum 25[OH]D are found in women with PPD compared to controls.
Robinson et al., 2014 [65] Australia Cohort study	To examine the association between maternal serum 25[OH]D at 18 weeks’ gestation and the likelihood of developing PPD symptoms in the first few days after birth in a cohort of Caucasian women enrolled in the Western Australian Pregnancy Cohort (Raine) Study. N = 796 <20 years = 72 20–29.9 years = 408 ≥30 years: 315	EPDS -1–5 symptoms -6+ symptoms	Liquid chromatography–tandem mass spectrometry <47 nmol/L (18 ng/mL) (quartile 1) 47–58 nmol/L (18–23.2 ng/mL) (quartile 2) 59–70 nmol/L (23.6–28 ng/mL) (quartile 3) >70 nmol/ L(28 ng/mL) (quartile 4) 25[OH]D deficiency: <20 ng/mL	(Quartile) = B coefficient (95% CI), *p*-value Binary logistic regression model [Postnatal depression symptoms—6+ symptoms] -Quartile 1: 2.19 (1.26–3.78). *p* = 0.006 -Quartile 2: 1.42 (0.80–2.54). *p* = 0.236 -Quartile 2: 1.52 (0.85–2.72). *p* = 0.158 -Quartile 3: 1 (Ref.) Multinomial logistic regression model [Postnatal depression symptoms. 1–5 symptoms] -Quartile 1: 1.43 (0.85–2.40). *p* = 0.182 -Quartile 2: 0.94 (0.57–1.55). *p* = 0.811 -Quartile 2: 1.10 (0.67–1.82). *p* = 0.696 - Quartile 3: 1 (Ref.) [Postnatal depression symptoms. 6+ symptoms] -Quartile 1: 2.72 (1.42–5.22). *p* = 0.003 -Quartile 2: 1.37 (0.71–2.63). *p* = 0.811 -Quartile 2: 1.61 (0.83–3.10). *p* = 0.158 -Quartile 3: 1 (Ref.) Adjusted for pre-pregnancy body mass index, maternal age, maternal education, total family income, maternal smoking, maternal alcohol intake, hypertensive diseases of pregnancy, proportion of optimal birth weight, and child gender.	Lower concentrations of 25[OH]D in the second trimester of pregnancy are linked to a greater risk for reporting postnatal depressive symptoms in the first days following birth.
Sağlam et al., 2021 [30] Turkey Cross-sectional study	To evaluate the relationship between depression, anxiety, sleep quality, and 25[OH]D deficiency in pregnant women. G1, 25[OH]D deficient (*n* = 90); G2, 25[OH]D sufficient (*n* = 63) G1 = 27.4 (5.7); G2 = 27.9 (5.9) years	BDI -Depression BDI 0–17: Without depression BDI >17: With depression -Anxiety BAI 8–15: mild anxiety BAI 16–25: moderate 26–63: severe anxiety	Immune chemiluminometric assay. NR	Depression—G1 vs. G2 BDI (0–17, without depression): 70 (77.8%); 63 (100%) BDI (>17, with depression): 20 (22.2%); 0 (0.0%) Anxiety—G1 vs. G2 Total: 15.3 (8.7) vs. NR BAI (8–15): 56 (6.2%); 61 (96.8%) BAI (16–25): 23 (25.6%); 2 (3.2%) BAI (≥26): 11 (12.2%); 0 (0.0%) Adjusted for NR.	Depression and anxiety during pregnancy were associated with 25[OH]D deficiency. The prevalence of depression/anxiety was significantly higher among mothers with 25[OH]D deficiency.
Yuvaci et al., 2020 [46] Turkey Cross-sectional	To evaluate the relationship between PPD and 25[OH]D concentrations in maternal blood and breast milk. N = 75 Mean age—29.80 (4.54) years G1—EPDS < 13: 30.13 (4.76) years G2—EPDS ≥ 13: 29.32 (4.21)	EPDS EPDS < 13: no depression EPDS ≥ 13: depression	Automated chemiluminescence immunoassay. The reportable range of the test is 7–125 ng/mL (18–133 nmol/L). Any value read below 7 ng/mL (18 nmol/L) was reported as “<7 ng/mL (18 nmol/L)”	Serum 25[OH]D: Mean (ng/mL) (SD), G1—16.87 (6.37), G2—17.54 (7.42) t, *p*-value. −0.676, *p* = 0.463 Adjusted for: NR.	No significant correlation was determined between the 25[OH]D concentrations in maternal blood and PPD.
Van Lee et al., 2020 [66] Singapore Cohort study	To investigate the cumulative risk of the lifestyle behaviors on depressive symptoms during pregnancy and after delivery. N = 535 EPDS ≥ 15 (*n* = 38)—28.4 (1.1) years; EPDS < 15 (*n* = 497)—30.8 (0.2) years	EPDS -EPDS ≥ 15: during pregnancy -EPDS ≥ 13: postpartum	Liquid chromatography–tandem mass spectrometry. Insufficient: <20 ng/mL.	25[OH]D (nmol/l) by depressive/non-depressive- 73.2 (3.8) vs. 81.7 (1.2)—*p* = 0.066 Adjusted for age, ethnicity, educational level, household income, employment status, and parity.	There were no differences detected between women with and without probable depression for 25[OH]D concentrations.
Wang et al., 2023 [67] China Cross-sectional study	To examine the association between 25[OH]D and PPD and AD in Chinese pregnant women and lactating women. Lactating women—N = 907; Pregnant women—N = 866 Pregnant women Non-AD: 29.2 (26.5, 32.1) years AD: 27.1 (25.7, 31.1) years	EPDS EPDS < 13: without PPD EPDS score ≥ 13: with PPD	Liquid chromatography–tandem mass spectrometry. Insufficient: <30 ng/mL Deficient: <20 ng/mL	Lactating women—PPD (AD) vs. Non-PPD (AD); Median (IQR) 25[OH]D—24.6 (18.9, 30.4); 26.2 (19.7, 33.2) (*p* = 0.034) 25[OH]D status (*p* = 0.064) <20 ng/mL: 48 (33.3%); 196 (25.7%) 20–30 ng/mL: 59 (41.0%); 304 (39.8%) ≥30 ng/mL: 37 (25.7%); 263 (34.5%) Pregnant women—AD vs. Non-AD; Median (IQR) 25[OH]D—26.2 (17.0, 36.9); 25.8 (17.1, 38.0) (*p* = 0.693) 25[OH]D status (*p* = 0.476) <20 ng/mL: 32 (37.6%); 257 (32.9%) 20–30 ng/mL: 18 (21.2%); 210 (26.9%) ≥30 ng/mL: 35 (41.2%); 314 (40.2%) Adjusted analysis PPD Sufficient: 1 (Ref.) Insufficient: 1.38 (0.89–2.15), *p* = 0.155 Deficient: 1.74 (1.09–2.78), *p* = 0.020 Continuous (per 5 ng/mL): 0.89 (0.80–0.99) (*p* = 0.038). AD Sufficient: 1 (Ref.) Insufficient: 0.68 (0.35–1.31), *p* = 0.246 Deficient: 1.02 (0.55–1.90), *p* = 0.954 Continuous (per 5 ng/mL): 1.00 (0.91–1.09) (*p* = 0.973). 25[OH]D deficiency was associated with a higher prevalence of PPD in lactating women (OR = 1.71, 95% CI: 1.01–2.88, *p* = 0.044). The serum 25[OH]D of lactating women was inversely associated with the scores of the factor “depressive mood” of the EPDS (OR per 5 ng/mL = 0.10, 95% CI: 0.19 to 0.01, *p* = 0.032). No significant association between serum 25-hydroxy25[OH]D and AD was observed. Adjusted for age, gestational period, education level, income, smoke status, alcohol status, work, preparation before pregnancy, physical activities, and sleep quality.	A significant association was observed between 25[OH]D status and PPD; however, the association between 25[OH]D status and AD was not significant.

Legend: 25[OH]D: vitamin D; 95% CI: 95% confidence interval; AD: antenatal depression; BDI: Beck Depression Inventory; CES-D: Center for Epidemiological Studies Depression; DASS-21: Depression, Anxiety, and Stress Scale; ELISA: enzyme-linked immunosorbent assay; EPDS: Edinburgh Postnatal Depression Scale; G: group; IQR: interquartile range; ng/mL: nanograms per milliliter; NR: not reported; OR: odds ratio; PASS: Perinatal Anxiety Screening Scale; PPD: postpartum depression; RR: risk ratio; SD: standard deviation; SE: standard error; STAI: State–Trait Anxiety Inventory; β: beta coefficient.

**Table 4:** Bibliometric analysis of the included studies.

Author Year	Bibliometric Data									
	Journal Impact Factor (2022)	CoI	ES	FS	CRT	AIS	Guidelines	Open Science		
								OA	PI	DS
Abedi et al., 2018 [52]	*The Journal of Medicine and Life* *NR*	Yes	Yes	NR	NR	No	No	Yes	No	No
Accortt et al., 2016 [54]	*Archives of Women’s Mental Health* 4.5	Yes	Yes	Yes	Yes	N/A	No	Yes	No	No
Accortt et al., 2021 [53]	*Journal of Women’s Health* 3.5	Yes	Yes	Yes	Yes	No	No	Yes	No	No
Al-Sabah et al., 2024 [43]	*Journal of Epidemiology and Global Health* 1.71	Yes	Yes	Yes	Yes	No	No	Yes	No	Yes
Basutkar et al., 2021 [45]	*International Journal of Pharmaceutical Research* NR	Yes	Yes	NR	NR	No	No	Yes	No	No
Brandenbarg et al., 2012 [55]	*Psychosomatic Medicine* 3.8	Yes	Yes	Yes	NR	NA	No	Yes	No	No
Cassidy-Bushrow et al., 2012 [56]	*Journal of Women’s Health* 3.5	NR	Yes	Yes	NR	NA	No	No	No	No
Desirée et al., 2024 [32]	*Psychosomatic Medicine* 4.31	Yes	Yes	Yes	Yes	No	No	Yes	No	Yes
Figueiredo et al., 2017 [47]	*Journal of Psychiatric Research* 4.8	NR	Yes	NR	NR	NA	No	No	No	No
Fu et al., 2015 [57]	*International Journal of Obstetrics & Gynaecology* 5.8	Yes	Yes	Yes	Yes	NA	No	No	No	No
Gould et al., 2015 [58]	*Australian and New Zealand Journal of Obstetrics and Gynaecology* 1.7	Yes	Yes	Yes	Yes	NA	No	No	No	No
Gur et al., 2014 [44]	*European Journal of Obstetrics & Gynecology and Reproductive Biology* 2.6	NR	Yes	NR	NR	NA	No	No	No	No
Hannan et al., 2023 [33]	*Archives of Women’s Mental Health* 4.5	Yes	Yes	Yes	NR	No	No	No	No	No
Huang et al., 2014 [31]	*Journal of Women’s Health* 3.5	Yes	Yes	Yes	NR	NA	No	Yes	No	No
King et al., 2022 [59]	*Reproductive Sciences* 2.9	Yes	Yes	Yes	Yes	No	No	No	No	Yes
Lamb et al., 2018 [60]	*Archives of Women’s Mental Health* 4.5	Yes	Yes	Yes	NR	No	No	No	No	No
Lin et al., 2019 [61]	*Nutrients* 5.9	Yes	Yes	Yes	Yes	NA	No	Yes	No	No
Murphy et al., 2010 [62]	*Journal of the American Psychiatric Nurses Association* 2.0	Yes	Yes	Yes	NR	NA	No	No	No	No
Nassr et al., 2022 [63]	*Middle East Current Psychiatry* 2.2	Yes	Yes	Yes	Yes	No	No	Yes	No	Yes
Pillai et al., 2021 [64]	*Archives of Medical Research* 7.7	Yes	Yes	Yes	NR	NR	No	No	No	No
Robinson et al., 2014 [65]	*Archives of Women’s Mental Health* 4.5	Yes	Yes	Yes	NR	NA	No	No	No	No
Sağlam et al., 2021 [30]	*Journal of Academic Research in Medicine* NR	Yes	Yes	Yes	Yes	NR	No	Yes	No	No
Yuvaci et al., 2020 [46]	*Journal of Clinical Obstetrics & Gynecology* 0.8	Yes	Yes	Yes	Yes	NR	No	Yes	No	No
Van Lee et al., 2020 [66]	*Comprehensive Psychiatry* 7.2	NR	Yes	Yes	Yes	NR	No	Yes	No	No
Wang et al., 2023 [67]	*Journal of Affective Disorders* 6.6	Yes	Yes	Yes	Yes	NR	No	Yes	No	No

Legend: AIS: artificial intelligence statement; CoI: conflict of interest; CRT: CRediT author statement; DS: data sharing; ES: ethical statement; FS: funding statement; NA: not applicable; NR: not reported; OA: open access; PI: protocol index.

## Data Availability

All the data and information can also be requested from the main author via email (adriano.assis@ucpel.edu.br), in accordance with the FAIR Data Principles.

## Supplementary Materials

The following supporting information can be downloaded at: https://www.mdpi.com/article/10.3390/nu16213648/s1, File S1. Search strategy by databases. File S2. (A) Certainty analysis of the evidence from all meta-analyses performed in this study. (B) Certainty analysis of the evidence from all meta-analyses performed in this study by status of Vitamin D (With deficient/insufficient *vs.* Without deficient/insufficient).
