# Supplementary material for: Is Serum Vitamin D Associated with Depression or Anxiety in Ante- and Postnatal Adult Women? A Systematic Review with Meta-Analysis
Source: Nutrients. 2024 Oct 26;16(21):3648. doi: 10.3390/nu16213648 (PMC11547481; doi:10.3390/nu16213648)
Supplement: Supplementary file 1 [file nutrients-16-03648-s001.zip › File S1.pdf]

File S1. Search strategy by databases.

N = 10.146

PubMed –2553 studies

#1 – Vitamin D[MeSH Terms] OR Vitamin D deficiency[MeSH Terms] OR Vitamin D[Text Word] OR 25-hydroxyvitamin D[Text Word] OR vitamins[MeSH Terms] OR vitamin[Text word] OR vitamins[Text word]

#2 – Anxiety[Mesh Terms] OR Anxiety[Text word] OR Anxiety Disorders[MeSH Terms] OR Depression[Mesh Terms] OR Depression[Text word] OR Mood disorders[Mesh Terms] OR Mood disorders[Text word] OR mental health[Mesh Terms] OR mental health[Text word] OR Depressive[Text word]

#3 – Women[Mesh Terms] OR Women[Text word] OR Postpartum Period[Mesh Terms] OR Maternal Health Services[Mesh Terms] OR Maternal Serum Screening Tests[Mesh Terms] OR Pregnancy[Mesh Terms] OR Pregnancy[Text word] OR Female[Mesh Terms] OR Female[Text word] OR Females[Text word] OR Girl[Text word] OR Postpartum[Text word]

#4 - #1 AND #2 AND #3

Scopus –2468 studies

((TITLE-ABS-KEY (vitamin AND d) OR TITLE-ABS-KEY (25-hydroxyvitamin AND d) OR TITLE-ABS-KEY (25-hydroxyvitamin) OR TITLE-ABS-KEY (vitamins) OR TITLE-ABS-KEY (vitamin))) AND ((TITLE-ABS-KEY (anxiety) OR TITLE-ABS-KEY ("Anxiety Disorders") OR TITLE-ABS-KEY (depression) OR TITLE-ABS-KEY ("Mood disorder") OR TITLE-ABS-KEY ("Mood disorders") OR TITLE-ABS-KEY ("mental health") OR TITLE-ABS-KEY (depressive))) AND ((TITLE-ABS-KEY (women) OR TITLE-ABS-KEY ("Postpartum Period") OR TITLE-ABS-KEY ("Maternal Health Services") OR TITLE-ABS-KEY ("Maternal Serum Screening Tests") OR TITLE-ABS-KEY (pregnancy) OR TITLE-ABS-KEY (postpartum)))

Web of Science –1724 studies

#1 – TS=(Vitamin D OR "Vitamin D deficiency" OR 25-hydroxyvitamin D OR vitamins OR vitamin)

#2 – TS=(Anxiety OR "Anxiety Disorder" OR "Anxiety Disorders" OR Depression OR "Mood disorders" OR "mental health" OR Depressive)

#3 – TS=(Women OR "Postpartum Period" OR "Maternal Health Services" OR "Maternal Serum Screening Tests" OR Pregnancy OR Female OR Females OR Girl OR Postpartum)

#4 - #1 AND #2 AND #3

Embase –2183 studies

#1 – (('vitamin'/exp OR vitamin) AND d OR 'vitamin d deficiency'/exp OR 'vitamin d deficiency' OR '25 hydroxyvitamin') AND d OR 'vitamins'/exp OR vitamins OR 'vitamin'/exp OR vitamin

#2 – 'anxiety'/exp OR anxiety OR 'anxiety disorders'/exp OR 'anxiety disorders' OR 'anxiety disorder'/exp OR 'anxiety disorder' OR 'depression'/exp OR depression OR 'mood disorders'/exp OR 'mood disorders' OR 'mood disorder'/exp OR 'mood disorder' OR 'mental health'/exp OR 'mental health' OR depressive

#3 – 'postpartum period'/exp OR 'postpartum period' OR 'maternal health services'/exp OR 'maternal health services' OR 'maternal serum screening test'/exp OR 'maternal serum screening test' OR 'pregnancy'/exp OR pregnancy OR 'postpartum'/exp OR postpartum

#4 – #1 AND #2 AND #3 AND [embase]/lim

Cochrane Library –1385 studies

#1 – Vitamin D OR "Vitamin D deficiency" OR 25 hydroxyvitamin D OR vitamins OR vitamin

#2 – Anxiety OR "Anxiety Disorder" OR "Anxiety Disorders" OR Depression OR "Mood disorders" OR "mental health" OR Depressive

#3 – Women OR "Postpartum Period" OR "Maternal Health Services" OR "Maternal Serum Screening Tests" OR Pregnancy OR Female OR Females OR Girl OR Postpartum

#4 - #1 AND #2 AND #3
