# Supplementary material for: Is Serum Vitamin D Associated with Depression or Anxiety in Ante- and Postnatal Adult Women? A Systematic Review with Meta-Analysis
Source: Nutrients. 2024 Oct 26;16(21):3648. doi: 10.3390/nu16213648 (PMC11547481; doi:10.3390/nu16213648)
Supplement: Supplementary file 1 [file nutrients-16-03648-s001.zip › File S2.pdf]

Supplementary File S2

S2(A). Certainty analysis of the evidence from all meta-analyses performed in this study.

**Assessment**	**Certainty assessment**							**№ of patients**		**Effect**		**Certainty**	**Importance**
**Outcome / exposure**	**№ of studies**	**Study design**	**Risk of bias**	**Inconsistency**	**Indirectness**	**Imprecision**	**Other considerations**	**With depression**	**Without depression**	**Relative (95% CI)**	**Absolute (95% CI)**		
Post-natal depression - Vitamin D measured in the postnatal	4	Non-RCT	Serious[a]	Not Serious	Not Serious	Not Serious	None	531	444	-	MD **2.36 ng/ml lower** (4.59 lower to 0.14 lower)	⊕◯◯◯ Very low	CRITICAL
Antenatal depression - Vitamin D measured during pregnancy	5	Non-RCT	Not Serious	Very Serious[b]	Not Serious	Serious[c]	None	1510	4135	-	MD **4.63 ng/ml lower** (8.88 lower to 0.34 lower)	⊕◯◯◯ Very low	CRITICAL

CI: confidence interval; MD: mean difference
a. Two studies presented a moderate/fair risk of bias (weighted as 67.1% for the whole analysis). Two other studies showed no risk of bias.
b. Very high heterogeneity was detected (I^2=91%).
c. A very high 95% confidence interval was detected.

Supplementary File 2

S2(B). Certainty analysis of the evidence from all meta-analyses performed in this study by status of Vitamin D (With deficient/insufficient *vs.* Without deficient/insufficient).

**Assessment**	**Certainty assessment**						**№ of patients**		**Effect**		**Certainty**	**Importance**
**Outcome / exposure**	**№ of studies**	**Study design**	**Risk of bias**	**Inconsistency**	**Indirectness**	**Imprecision**	**Other considerations**	**With deficient / insufficient Vitamin D**	**Without deficient / insufficient Vitamin D**	**Relative (95% CI)**	**Absolute (95% CI)**		
Postnatal depression - Vitamin D measured postnatal (Vitamin D <30 ng/ml) – Crude analysis	3	Non-RCT	Serious [a]	Serious [b]	Not Serious	Very Serious [c]	None	336/622 (54.0%)	85/223 (38.1%)	**OR 1.70** (0.76 to 3.82)	**130 more per 1.000** (from 62 fewer to 321 more)	⊕◯◯◯ Very low	CRITICAL
Postnatal depression - Vitamin D measured postnatal (vitamin D <20 ng/ml) - Crude analysis	3	Non-RCT	Serious [d]	Serious [e]	Not Serious	Serious [f]	None	185/347 (53.3%)	236/508 (46.5%)	**OR 1.60** (0.63 to 4.08)	**117 more per 1.000** (from 111 fewer to 315 more)	⊕◯◯◯ Very low	CRITICAL
Postnatal depression - Vitamin D measured postnatal (Vitamin D <20	2	Non-RCT	Very Serious [g]	Not Serious	Not Serious	Not Serious	Strong association all plausible residual confounding would reduce			**OR 2.13** (1.16 to 3.90)	**2 fewer per 1.000** (from 4 fewer to 1 fewer)	⊕⊕◯◯ Low	CRITICAL

ng/ml) - Adjusted analysis							the demonstrated effect.						
Postnatal depression - Vitamin D measured during pregnacy (Vitamin D <30 ng/ml)	2	Non-RCT	Very Serious[g]	Not Serious	Not Serious	Very Serious[h]	None	44/260 (16.9%)	8/26 (30.8%)	**OR 0.48** (0.20 to 1.17)	**132 fewer per 1.000** (from 226 fewer to 34 more)	⊕◯◯◯ Very low	CRITICAL
Depression during pregnacy - Vitamin D measured during pregnacy (Vitamin D <30 ng/ml)	3	Non-RCT	Not Serious	Not Serious	Not Serious	Very Serious[i]	None	283/1112 (25.4%)	51/312 (16.3%)	**OR 1.51** (0.98 to 2.30)	**64 more per 1.000** (from 3 fewer to 147 more)	⊕◯◯◯ Very low	CRITICAL
Depression during pregnacy - Vitamin D measured during pregnacy (Vitamin D <20 ng/ml)	4	Non-RCT	Serious[j]	Very Serious[k]	Not Serious	Very Serious[l]	None	225/859 (26.2%)	129/718 (18.0%)	**OR 2.16** (0.74 to 6.37)	**141 more per 1.000** (from 40 fewer to 403 more)	⊕◯◯◯ Very low	CRITICAL

Legend: **CI:** confidence interval; **OR:** odds ratio; **non-RCT**: non-randomized clinical trials

a. Two studies presented moderate/fair risk of bias (weighted as 48.8% for the whole analysis).

b. Heterogeneity was high (I^2=60%).

c. No statistically significant association between groups. The outcome was detected in only 421 women.

d. Two studies presented moderate/fair risk of bias (weighted as 53.8% for the whole analysis).

e. Heterogeneity was high (I^2=75%).

f. No statistically significant association between groups.

g. All studies included in this analysis presented risk of bias.

h. No statistically significant association between groups. The outcome was detected in only 52 women.

i. No statistically significant association between groups. The outcome was detected in only 332 women.

j. One study presented moderate/fair risk of bias (weighted as 10.4% for the whole analysis).

k. Heterogeneity was very high (I^2=78%).

l. No statistically significant association between groups. The outcome was detected in only 354 women.
